# Rhenium(I) tricarbonyl complexes with 1,10-phenanthroline derivatives: potential anticancer agents for hepatocellular carcinoma

**DOI:** 10.3389/fphar.2026.1926840

**Published:** 2026-09-21

**Authors:** César Zúñiga, Diego Oyarzún, Carlos Cardoso, Valentina Melo, Ignacio Montorfano, Yolanda Prado, Felipe Simon, Juan F. Santibáñez, Rodrigo Ramírez-Tagle, Javier Echeverría, César Echeverría

**Affiliations:** 1 Instituto de Ciencias Naturales, Facultad de Medicina Veterinaria y Agronomía, Universidad de Las Américas, Santiago, Chile; 2 Centro de Investigación en Ciencias Biológicas y Químicas, Universidad de Las Américas, Santiago, Chile; 3 Departamento de Química y Biología, Facultad de Ciencias Naturales, Universidad de Atacama, Copiapó, Chile; 4 ATACAMA-OMICS, Laboratorio de Biología Molecular y Genómica, Facultad de Medicina, Universidad de Atacama, Copiapó, Chile; 5 Center for Research on Pandemic Resilience, Faculty of Life Sciences and Institute of Public Health, Universidad Andres Bello, Santiago, Chile; 6 Millennium Institute on Immunology and Immunotherapy, Santiago, Chile; 7 Centro Integrativo de Biología y Química Aplicada (CIBQA), Universidad Bernardo O'Higgins, Santiago, Chile; 8 Group for Molecular Oncology, University of Belgrade, Institute for Medical Research, National Institute of Republic of Serbia, Belgrade, Serbia; 9 Facultad de Ingeniería y Arquitectura, Universidad Central de Chile, Santiago, Chile; 10 Departamento de Ciencias del Ambiente, Facultad de Química y Biología, Universidad de Santiago de Chile, Santiago, Chile

**Keywords:** apoptosis, caspases, cytotoxicity, hepatocellular carcinoma, rhenium complexes

## Abstract

**Background:**

Hepatocellular carcinoma (HCC) is a highly lethal malignancy with limited therapeutic options for advanced disease, motivating the development of metal-based agents that can bypass resistance and improve tumor selectivity.

**Purpose:**

This study aimed to evaluate rhenium(I) tricarbonyl complexes bearing 1,10-phenanthroline derivatives, with emphasis on *fac*-[Re(CO)_3_(5-chloro-1,10-phenanthroline)Cl] (Re(3)), as potential anti-HCC candidates and to elucidate mechanistic determinants of activity in HepG2 cells.

**Materials and Methods:**

Complexes *fac*-[Re(CO)_3_(NH_2_phen)Cl] (Re(1)), *fac*-[Re(CO)_3_(NO_2_phen)Cl] (Re(2)), and Re(3) were synthesized under argon and characterized by ^1^H NMR; electronic structure was examined by DFT (ZORA-PW91/TZP) with COSMO (water), including global reactivity descriptors and frontier orbital composition. Antiproliferative effects were quantified in HepG2 and non-tumor Vero cells using a 72 h resazurin assay (1–150 μM), while apoptosis, caspase activity, oxidative stress, and signaling changes were assessed by Annexin V/7-AAD flow cytometry, Caspase-Glo 3/7, DCFH-DA ROS measurements, western blotting, and immunofluorescence microscopy; statistics used Kruskal–Wallis/Dunn or ANOVA with *p* < 0.05.

**Results and Discussion:**

Re(3) showed pronounced, selective cytotoxicity (IC_50_ 6 ± 1.1 μM at 72 h; selective index 5.7; Vero IC_50_ > 30 μM), whereas Re(1) and Re(2) were largely inactive (IC_50_ > 100 μM). Re(3) induced predominantly apoptotic death (>60% apoptotic cells at 48 h; minimal necrosis), increased caspase-3/7 activity (4–16 h), triggered an early ROS burst (1–20 μM; significant at 2 and 4 h, comparable to CuSO_4_), upregulated cytochrome c, caspase-9 and caspase-3, downregulated Bcl-XL, and increased phosphorylated p53, with negligible extrinsic signaling via caspase-8. DFT indicated Re(3) has a stabilized LUMO (−3.34 eV) and electrophilicity (ω = 4.32 eV) with an “optimal” hardness (η = 2.37 eV) supporting sufficient redox reactivity yet kinetic stability; the LUMO localizes (>90%) on the chloro-phenanthroline, consistent with MLCT-driven redox stress.

**Conclusion:**

Collectively, Re(3) functions as a tuned redox-active agent, triggering apoptosis through the intrinsic pathway. This process possibly involves mitochondrial modulation with tumor selectivity, supporting its advancement as a lead rhenium-based prototype for HCC and motivating *in vivo* pharmacology and broader HCC panel validation.

## 1 Introduction

Hepatocellular carcinoma (HCC) is the most prevalent primary malignancy of the liver and represents one of the leading causes of cancer-related mortality worldwide. It accounts for approximately 90% of all liver cancer cases, with an estimated incidence exceeding 800,000 new diagnoses per year. The major etiological factors include chronic hepatitis B (HBV) and hepatitis C (HCV) infections, excessive alcohol consumption, aflatoxin B_1_ exposure, obesity, and non-alcoholic fatty liver disease. These risk factors converge on chronic hepatic inflammation, oxidative stress, and genomic instability, leading to cirrhosis and hepatocellular malignant transformation. At a molecular level, HCC progression is characterized by dysregulation of key signaling pathways, including p53, PI3K/AKT/mTOR, MAPK, and Wnt/β-catenin. These pathways collectively promote cell survival, angiogenesis, and resistance to apoptosis (Szliszka et al., 2009; Leonidova and Gasser, 2014). Despite advances in diagnostic imaging and targeted therapies, the overall five-year survival rate for advanced HCC remains below 20%, underscoring the urgent need for novel therapeutic strategies that can selectively target cancer cells while minimizing systemic toxicity.

Transition metal complexes have emerged as an essential class of compounds in anticancer drug development due to their structural diversity and ability to interact with biological macromolecules. Following the clinical success of platinum-based drugs such as cisplatin and oxaliplatin, attention has shifted toward other transition metals that can overcome limitations associated with platinum therapy, including resistance mechanisms, nephrotoxicity, and poor selectivity (Leonidova and Gasser, 2014). Among these, rhenium (Re) complexes have attracted growing interest for their unique photophysical, redox, and coordination properties (Frin and Murakami Iha, 2011; Palion-Gazda et al., 2024).

Rhenium typically forms stable complexes in oxidation states 1+ and 3+, and its *fac*-[Re(CO)_3_]^+^ core provides a robust and tunable platform for ligand substitution. This structural versatility allows fine control over lipophilicity, cellular uptake, and subcellular localization.

From a biological perspective, rhenium complexes exhibit multimodal anticancer mechanisms. They can induce mitochondrial modulation, generate reactive oxygen species (ROS), and activate intrinsic apoptotic pathways (Echeverría et al., 2012). In hepatocellular carcinoma cells, these processes can trigger cytochrome c release, caspase-9 activation, and subsequent caspase-3 cleavage, resulting in programmed cell death (Delasoie et al., 2020).

Rhenium compounds have also been shown to interact with DNA and nuclear proteins, causing double-strand breaks and modulation of transcription factors such as p53 and NF-κB (Leonidova and Gasser, 2014). Unlike platinum drugs, many Re(I) tricarbonyl complexes act via redox modulation rather than direct DNA crosslinking, potentially reducing genotoxic side effects(Marker et al., 2018). Their ligand environment influences the biological fate of rhenium compounds. Complexes containing 1,10-phenanthroline (phen) and its derivatives display remarkable cytotoxicity due to their planarity and capacity for π–π stacking with nucleic acids (Frin and Murakami Iha, 2011; Świtlicka et al., 2016).

Substitution on the phenanthroline ring—such as halogenation (e.g., 5-chloro-phenanthroline)—enhances lipophilicity, cellular uptake, and mitochondrial accumulation (Palion-Gazda et al., 2024). Once the cells internalize these complexes, they can localize within mitochondria and lysosomes, disrupting membrane potential and promoting oxidative stress (Knopf et al., 2017).

Furthermore, rhenium complexes can exploit photoactivation to generate singlet oxygen or release carbon monoxide, adding a level of layer of cytotoxic control through photodynamic therapy mechanisms (Marker et al., 2018).

In addition to their therapeutic potential, rhenium isotopes such as ^188^Re and ^186^Re possess favorable radiochemical properties for theranostic applications. Their β^−^ emissions enable targeted radiotherapy, while their accompanying γ-emission allows imaging and biodistribution (Leonidova and Gasser, 2014).

This dual functionality makes rhenium an attractive element for designing agents that combine diagnosis and therapy, particularly relevant in the treatment of liver tumors accessible via intra-arterial delivery.

Rhenium(I) tricarbonyl complexes with substituted 1,10-phenanthroline ligands have been studied for several applications. These studies include luminescent molecules (Frin and Murakami Iha, 2011; McGhie and Aldrich-Wright, 2022) and compounds for anion detection (Ng et al., 2011; Bessette et al., 2012), electronic properties (Gabrielsson et al., 2005; Hu et al., 2017; Gonçalves et al., 2019), electrochemical characteristics (Bullock et al., 2008), photophysical attributes (Bare et al., 2004; Zhao et al., 2012; Ray and Rajak, 2025), and non-linear optical (NLO) features (Sanhueza et al., 2013). In the medical field, Re(I) tricarbonyl complexes have garnered considerable attention for their potential as anticancer agents, particularly through structural designs incorporating ligands such as 1,10-phenanthroline derivatives (Leonidova and Gasser, 2014; Konkankit et al., 2019; Marker et al., 2020). Recent studies have revealed unexpectedly high cytotoxicity of certain tricarbonyl rhenium complexes bearing imidazo[4,5-f]1,10-phenanthroline derivatives, thereby demonstrating potential anticancer applications (Enslin et al., 2023; Babu et al., 2024). When accumulated in cellular organelles, these complexes can induce different cell death pathways, including apoptosis and paraptosis, while maintaining activity under hypoxic conditions (Enslin et al., 2023; de Lavor et al., 2024). Moreover, prior literature has highlighted the merits of *fac*-[Re(CO)_3_]^+^ scaffolds in chemotherapeutic approaches, underscoring how structural variations in the ancillary ligands can influence antiproliferative efficacy (Komreddy et al., 2021; Palion-Gazda et al., 2024). Previous research has shown that the structures of rhenium complexes enable enhanced cytotoxicity and mechanisms of action, making them potential anticancer agents. Re(I) tricarbonyl complexes carrying 1,10-phenanthroline derivatives often rely on metal-to-ligand charge transfer (MLCT) and intraligand (IL) transitions that promote distinct photophysical and photochemical behaviors relevant to therapeutic or imaging applications (Komreddy et al., 2021; Choroba et al., 2023; Palion-Gazda et al., 2024). Substituents on the phenanthroline ring can adjust the MLCT energies, redox potentials, and excited-state lifetimes of the complexes (Kurtz et al., 2015). Various electron-donating or electron-withdrawing groups can either enhance luminescence efficiency or induce effective DNA/protein binding, both of which are closely linked to tumor cell viability (Świtlicka et al., 2016; Palion-Gazda et al., 2024). On the other hand, have also synthesized derivatives bearing additional functional groups to increase water solubility, improve targeting, or facilitate specific modes of cell death (Marker et al., 2018; Jalilehvand et al., 2023). Enhancing solubility, for example, can improve biodistribution profiles, thereby affecting overall therapeutic outcomes (Knopf et al., 2017). Therefore, in designing *fac*-[Re(CO)_3_] complexes for cancer therapy, the chelating diimine framework—especially 1,10-phenanthroline—confers both photochemical stability and reactivity, which are harnessed through chemical substitutions (Frin and Murakami Iha, 2011; Komreddy et al., 2021). Mechanistic insights into Re(I)-based anticancer activity often point to multiple pathways. Specific complexes show potent cytotoxicity against cancer cells through direct DNA binding, mitochondrial disruption, or interference with metabolic processes (Delasoie et al., 2020; Enslin et al., 2023).

Studies have reported that some Re(I) tricarbonyl compounds induce cell death pathways distinct from classical apoptosis, including caspase-independent cell death and paraptosis-like events (Ye et al., 2016; Knopf et al., 2017; Enslin et al., 2023). Specifically, the Re(I) tricarbonyl aqua complex family reported by Knopf and co-workers triggered cytoplasmic vacuolization and a cell-death mode that did not conform to canonical apoptosis, necrosis, paraptosis, or autophagy, while remaining fully active in cisplatin-resistant HeLa cells (Knopf et al., 2017). Subsequent work by Ye et al. demonstrated that subtle variation in lipophilicity between closely related phosphorescent mononuclear and dinuclear Re(I) complexes governs their subcellular localization—driving accumulation either in lysosomes, where it induced caspase-independent apoptosis, or in mitochondria, where it induced caspase-independent paraptosis (Ye et al., 2016).

Enslin and co-workers later showed that 1,10-phenanthroline-derived Re(I) tricarbonyl aqua complexes can reach very low nanomolar cytotoxicity in PC3 prostate cancer cells while retaining up to 26-fold selectivity over normal RPE-1 cells, with paraptosis again emerging as a discrete death pathway for the mitochondrial-localized analog (Enslin et al., 2023). Furthermore, the recent report on dinuclear Re(I) tricarbonyl complexes establishes that increasing nuclearity is a viable strategy for enhancing anticancer potency. These complexes are internalized via energy-dependent endocytosis, accumulate predominantly in mitochondria, induce apoptotic cell death across a panel of cancer cell lines at micromolar concentrations, and—most strikingly—eradicate a pancreatic carcinoma multicellular tumor spheroid at micromolar concentrations (Montesdeoca et al., 2024).

In photoactivation or photosensitization, Re(I) complexes can exploit light-induced reactions to generate ROS or release carbon monoxide, thereby inducing cytotoxic effects (Marker et al., 2018). This versatility stems from the inherently robust photophysics of *fac*-[Re(CO)_3_]^+^ cores (Frin and Murakami Iha, 2011; Choroba et al., 2023). Beyond photodynamic contexts, a 2024 study of twelve Re (I) tricarbonyl complexes prepared in a “2 + 1” coordination mode from deprotonated 8-hydroxyquinolines and a series of diazoles reported IC_50_ values of 1.5–14 µM against HL-60 promyelocytic leukemia cells together with measurable selectivity toward MCF-7 breast cancer cells relative to non-cancerous MCF-10A cells, consolidating the case for the 8-hydroxyquinoline rhenium scaffold as a privileged anticancer motif (Łyczko et al., 2024).

The selectivity of Re(I) complexes highlights their greater toxicity toward malignant cells than toward normal cells (Enslin et al., 2023). This distinction may be partly attributed to differential uptake routes, increased uptake to meet mitochondrial ATP requirements in rapidly proliferating cells, or enhanced reactivity with intracellular targets. Multiple studies have shown potent *in vivo* tumor inhibition at relatively low doses, indicating both efficacy and an acceptable safety window (Konkankit et al., 2019). For instance, Re(I) compounds tested in ovarian cancer xenograft models demonstrated significant suppression of tumor growth, mirroring or exceeding that of established platinum-based agents. Our focus on Re(I) organometallic complexes in biological assays, specifically tricarbonyl complexes derived from 1,10-phenanthroline, aligns with observations that these molecules can exhibit potent antiproliferative activities, improved selectivity, and versatile photophysical properties (Enslin et al., 2023). Adjusting the phenanthroline substituents modulates both electronic and steric factors that are critical for DNA binding, mitochondrial accumulation, and the generation of cytotoxic intermediates (Frin and Murakami Iha, 2011; Palion-Gazda et al., 2024). By combining the robust *fac*-[Re(CO)_3_]^+^ core with tailored phenanthroline-based ligands, it is possible to develop novel anticancer Re(I) anticancer agents that may outperform or complement existing metal-based chemotherapies.

The complex *fac*-[Re(CO)_3_(Clphen)Cl], containing the 5-chloro-1,10-phenanthroline ligand, represents a promising prototype for exploring these biological interactions. Its design integrates the photochemical robustness of the Re(I) tricarbonyl scaffold with the electronic modulation of a halogenated diamine ligand, hypothesized to enhance cytotoxic selectivity toward HCC cells. The present study aims to evaluate the cytotoxic, apoptotic, and oxidative stress responses induced by this compound in HepG2 cells, providing insight into the molecular mechanisms underlying its anticancer activity and supporting its potential development as a rhenium-based chemotherapeutic agent for liver cancer.

## 2 Materials and methods

### 2.1 Experimental section

All reactions were carried out under an argon atmosphere. Organic solvents were dried using standard procedures prior to use. [Re(CO)_5_Cl], 5-amino-1,10-phenanthroline, 5-nitro-1,10-phenanthroline, and 5-chloro-1,10-phenanthroline were purchased from Aldrich and used without further purification. ^1^H-NMR spectra were acquired on a Bruker AVANCE 400 MHz spectrometer (Supplementary Figures S1–S3). All chemical shifts are reported in parts per million (ppm) relative to tetramethylsilane (TMS) as the internal standard. Infrared spectra were recorded using a PerkinElmer FTIR “Spectrum Two” spectrometer equipped with a UATR accessory. Samples were placed directly on the diamond crystal, pressed to 30% of the total supported pressure, and scanned over the range of 4,000–500 cm^-1^ with a resolution of 1 cm^-1^ (Supplementary Figures S4–S6). The following rhenium complexes were synthesized according to previously reported procedures: *fac*-[Re(CO)_3_(NH_2_phen)Cl] (Re(1)), *fac*-[Re(CO)_3_(NO_2_phen)Cl] (Re(2)), and *fac*-[Re(CO)_3_(Clphen)Cl] (Re(3))(Frin and Murakami Iha, 2011; Sanhueza et al., 2013; Kurtz et al., 2015) (Figure 1).

**FIGURE 1:**
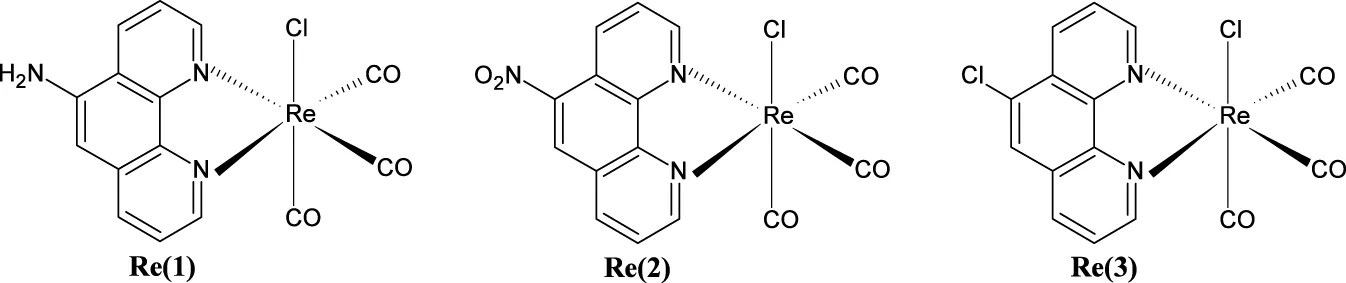
Rhenium(I) complexes of this study.

### 2.2 Computational details

Density Functional Theory (DFT) calculations were performed using the ADF 2024 program code. The molecular structures of the rhenium complexes were fully optimized without symmetry constraints using the Generalized Gradient Approximation (GGA) PW91 functional. The electronic configuration was described using TZP Slater-type basis sets, and scalar relativistic effects were treated via the ZORA Hamiltonian. Solvation effects were modeled using COSMO (water) to simulate the physiological environment. Global reactivity descriptors (ω, μ, and η) were calculated from the frontier orbital energies obtained in the solution phase.

### 2.3 Cell culture

The HCC cell line HepG2 and non-transformed Vero cells (American Type Culture Collection, ATCC-HB-8065 and ATCC-CCl-81) were cultivated in a monolayer format using Dulbecco’s modified Eagle Medium (DMEM) antibiotic-antimycotic (Gibco, NY, United States). The cultivation was conducted at 37 °C within a humidified incubator containing 5% CO_2_.

### 2.4 Cell viability assays

In the cell viability experiments, each cell type was seeded into 96-well plates at 5 × 10^3^ cells per well 24 h before the start of the experiment, before the addition of the stimulus compounds. A 10% solution of dimethyl sulfoxide (DMSO) was used as a positive control to induce cell death, whereas cells exposed only to DMEM supplemented with 1% fetal bovine serum (FBS) served as a negative control. The stimulus compounds were diluted in DMEM supplemented with 1% FBS from a 100 mM stock solution in DMSO (Merck, Germany) to produce different concentrations (1–150 μM) of Re(I) compounds. After 24, 48, and 72 h, the impact of the treatments on cell viability was assessed using the resazurin fluorometric assay. Resazurin salt, a blue N-oxide derivative of the fluorescent dye resorufin, is effective in detecting cellular reductive activity, making it suitable for evaluating cell proliferation and mitochondrial metabolic function. Each well received 20 μL of a 0.5 mg/mL resazurin solution in phosphate-buffered saline (PBS) and was incubated for 4 h under standard conditions. Subsequently, fluorescence was measured at an excitation wavelength of 560 nm and an emission wavelength of 590 nm using a 96-well microplate reader (Tecan Infinite® 200 Pro, Männedorf, Switzerland). The IC_50_ values were calculated using GraphPad Prism 10.

### 2.5 Annexin-V apoptosis assay

HepG2 cells were seeded at 5 × 10^5^ cells per well in a 6-well plate to reach approximately 70%–80% confluency in 24–48 h. Then, treatment with 2 μM Doxorubicin (DOX) for 24 h and 5 μM of **Re(3)** for 24 and 48 h. Apoptosis was assessed using flow cytometry. Phosphatidylserine expression on apoptotic cells was evaluated using fluorescein-labeled Annexin V conjugate and a viability co-stain like 7-AAD according to the eBioscience™ Annexin V Apoptosis Detection Kits protocol (Life Technologies, CA, United States). Analysis was performed on a BD Accuri C6 flow cytometer, with 10 × 10^4^ events per sample.

### 2.6 Caspase assays

The Caspase-Glo 3/7 assay (Promega, Madison, WI, United States) was used to measure the activity of the key effector caspases 3 and 7. Cells were seeded at a density of 2 × 10^3^ cells/well in 96-well white-walled plates. The next day, cells were treated with 25 μM of Re(3) compound. Cleaved caspase 3/7 activity was assessed at 4 and 8 h. Caspase-Glo reagent was added to the wells, mixed, and incubated in the dark at room temperature for 90 min. Luminescence signals were measured using a microplate reader (Tecan infinite® M200 Pro, Männedorf, Switzerland). Each treatment was read in at least four replicates.

### 2.7 Determination of intracellular ROS

2,7-Dichlorodihydrofluorescein diacetate (DCFH-DA) is a cell-permeable probe used to detect total intracellular ROS levels. Inside the cell, it is converted to DCF-DA by esterases, and then rapidly oxidized to form highly fluorescent DCF, reflecting total intracellular ROS levels (Leonidova and Gasser, 2014). Cells were seeded at a density of 5 × 10^3^ cells/well in 96-well black-walled plates. The following day, cells were exposed to 10 μM DCFH-DA for 30 min, after which they were rinsed with PBS. Subsequently, the cells were treated with Re(3) compound at concentrations ranging from 1 to 20 μM for a duration of 2–4 h. DCF fluorescence was measured at an excitation wavelength of 503 nm and an emission wavelength of 523 nm using a 96-well microplate reader (Tecan infinite® M200 Pro, Männedorf, Switzerland). The total intracellular ROS levels were expressed as DCF relative fluorescence units (RFU).

### 2.8 Western blot

HepG2 cells were seeded at 5 × 10^5^ cells per well in a 6-well plate to reach approximately 70%–80% confluency in 24–48 h. Then, treatment with 20 μM of Re(3) for 0.5–8 h was applied, and the cells were lysed using a cold lysis buffer (Tris-HCl, NaCl, EDTA, Triton X-100, protease and phosphatase inhibitor cocktail). The lysis conditions (ice-cold, 30 min, with intermittent vortexing) were followed by protein extraction. The protein extract was subjected to Sodium Dodecyl Sulfate Polyacrylamide Gel Electrophoresis (SDS-PAGE), and the separated proteins were transferred onto a nitrocellulose or polyvinylidene fluoride (PVDF) membrane. After blocking, the membrane was incubated with the appropriate primary antibody, washed twice, and then treated with a secondary antibody. Bands were visualized using a peroxidase-linked IgG antibody. Tubulin and α-actin were used as loading controls. A comprehensive list of the antibodies used is provided in Supplementary Table S1.

### 2.9 Fluorescence microscopy

HepG2 cells were seeded at 5 × 10^4^ cells per well in a Nunc^TM^ Lab-Tek^TM^ plate (Life Technologies, CA, United States) to reach approximately 70%–80% confluency in 24–48 h. Then, treatment with 5 μM of Re(3) for 24 h was applied. Cells were rinsed twice with PBS before being fixed (4% paraformaldehyde, 15 min at room temperature). The cells were rewashed and incubated with primary antibodies. Subsequently, the cells were washed twice and incubated with secondary antibodies. The samples were mounted using ProLong Gold antifade mounting medium with 4′,6-diamidino-2-phenylindole (DAPI) (Invitrogen, CA, United States). For a comprehensive list of antibodies used, refer to Supplementary Table S2. Fluorescent cells were examined using the EVOS® FLoid® cell imaging system (Life Technologies, CA, United States).

### 2.10 Data analysis

In all experiments, replicates were made, and the data presented represent the mean ± standard error (SEM) of at least three independent experiments performed in triplicate. Comparisons were conducted using a one-way analysis of variance (ANOVA) (Kruskal–Wallis), followed by Dunn’s *post hoc* test, and considered significant at *p* < 0.05.

## 3 Results

### 3.1 Computational insights into the mechanism of action

#### 3.1.1 Electronic structure and redox potential

Structural characterization of new compounds is a valid step in the contemporary search for potent anticancer agents. We performed DFT calculations incorporating solvation effects (COSMO: water) to mimic the cytosolic environment. The calculated electronic descriptors (Table 1) reveal that the substitution pattern on the phenanthroline ligand critically modulates the complexes’ basal redox properties.

**TABLE 1:** Electronic descriptors calculated at the ZORA-PW91/TZP level with COSMO solvation (water).

Compound	Substituent (R)	HOMO (eV)	LUMO (eV)	H-L gap (ΔE, eV)	Chemical potential (μ, eV)	Electrophilicity index (ω, eV)
Re(1)	−NH_2_	−5.44	−3.23	2.22	−4.33	4.24
Re(2)	−NO_2_	−5.74	−4.08	1.66	−4.91	7.25
Re(3)	−Cl	−5.72	−3.34	2.37	−4.53	4.32

The active complex Re(3) exhibits a stabilized Lowest Unoccupied Molecular Orbital (LUMO) energy of −3.34 eV, significantly lower than that of the inactive amino-substituted analog Re(1) (−3.23 eV). This stabilization implies a more favorable thermodynamic driving force for electron acceptance at the molecular level. Furthermore, the global electrophilicity index (ω) confirms this trend: Re(3) (ω = 4.32 eV) acts as a stronger electrophile compared to Re(1) (ω = 4.24 eV). These intrinsic electronic descriptors indicate that the chlorinated complex is electronically primed to accept electrons, a fundamental property for molecules capable of participating in redox cycling and ROS generation.

Interestingly, the nitro-substituted analog Re(2) displays the highest electrophilicity (ω = 7.25 eV) and the smallest HOMO-LUMO gap (1.66 eV). While theoretically the most potent electron acceptor of the series, these DFT-derived values are strictly intrinsic molecular descriptors. They reflect the basal reactivity of the isolated complexes but cannot account for the complex physiological factors—such as intracellular stability, protein binding affinity, or *in vivo* pharmacokinetics—that ultimately govern biological efficacy.

Analysis of the chemical hardness (η), defined as half of the HOMO-LUMO gap, provides further insight into the electronic configuration of the complexes. Re(3) possesses the highest chemical hardness (η = 2.37 eV) of the series, surpassing both the amino (η = 2.22 eV) and nitro (η = 1.66 eV) derivatives. According to the principle of maximum hardness, a larger energy gap corresponds to a less polarizable, more chemically stable electron cloud. The extremely small gap of the nitro complex (Re(2)) renders it a chemically “soft” and highly reactive species. While these computational descriptors successfully establish the fundamental electronic profiles—highlighting Re(3) as a stable electrophile—the translation of these intrinsic properties into pharmacological activity remains subject to the dynamic cellular environment.

#### 3.1.2 Orbital topology and charge localization

The rational design strategy is further validated by the population analysis of the frontier orbitals (Table 2) and the corresponding electron density maps (Supplementary Figure S7). As depicted in the frontier orbital plots for the active complex (HOMO Re(3) and LUMO Re(3)), the Highest Occupied Molecular Orbital (HOMO) is predominantly localized on the Rhenium center and the axial chloride ligand, consistent with a metal-ligand antibonding character. Conversely, the LUMO is almost exclusively localized (>90%) on the 5-chloro-1,10-phenanthroline ligand. A comparable orbital distribution is observed for the inactive derivative (HOMO Re(2) and LUMO Re(2)), albeit with different energetic stabilizations. This clear separation of charge density confirms the MLCT nature of the excited states and identifies the chlorinated phenanthroline moiety as the specific “landing pad” for incoming electrons. The electron-withdrawing effect of the chlorine atom stabilizes this π* orbital without altering the metal-centered oxidation potential, effectively tuning the intrinsic electronic properties of the complex.

**TABLE 2:** Percentage contributions based on Symmetrized Fragment Orbitals (SFOs) analysis.

MO	Character	Contribution from Re (%)	Contribution from axial Cl/CO (%)	Contribution from phen ligand (%)
HOMO	*dπ*(Re)/*π*(Cl)	55	25	20
LUMO	*π∗*(Phen)	<5	<5	>90

### 3.2 Cytotoxicity of rhenium complexes in HepG2 cells

The cytotoxic potential of rhenium complexes, Re(1), Re(2), and Re(3) (Figure 1), was evaluated on HepG2 (HCC) and Vero (non-tumor) cells at 24, 48, and 72 h. Among the tested species, the Re(I) tricarbonyl complex Re(3) displayed the most pronounced and selective cytotoxicity against HepG2 cells in a dose-dependent manner, with an IC_50_ of 6 ± 1.1 μM at 72 h and a selectivity index of 5,7 with limited toxicity in Vero cells (>30 μM). In contrast, Re(1) and Re(2) complexes showed negligible cytotoxic effects (IC_50_ > 100 μM), indicating that oxidation state and ligand environment critically influence antitumor activity (Table 3, Figure 2).

**TABLE 3:** *In vitro* cytotoxicity of rhenium complexes, Re(1), Re(2), and Re(3), on HepG2 cell lines and Vero cells, and the selective index values are shown in parentheses.

Compounds	IC_50_ values^a^ (μM)					
	24 h		48 h		72 h	
	Vero	HepG2	Vero	Hep-G2	Vero	Hep-G2
Re(1)	>100	>100	>100	>100	>100	>100
Re(2)	25.1 ± 1.1	39.5 ± 1.1	62.1 ± 1.4	43.5 ± 1.1	>100	>100
Re(3)	44.2 ± 2.2	26.3 ± 1.6(1.7)^b^	40.1 ± 8.4	11.6 ± 1.8(3.4)^b^	34.1 ± 6.9	6 ± 1.1 (5.7)^b^

^a^ IC_50_ (μM) values were determined in three independent experiments, each performed in triplicate, over the range of 0.05–100 μM.

^b^ Selective index = IC_50_ value on Vero cell/IC_50_ value on HepG2 cell line, in bold and in parentheses. Data shown are mean ± SEM, of 3 independent experiments.

**FIGURE 2:**
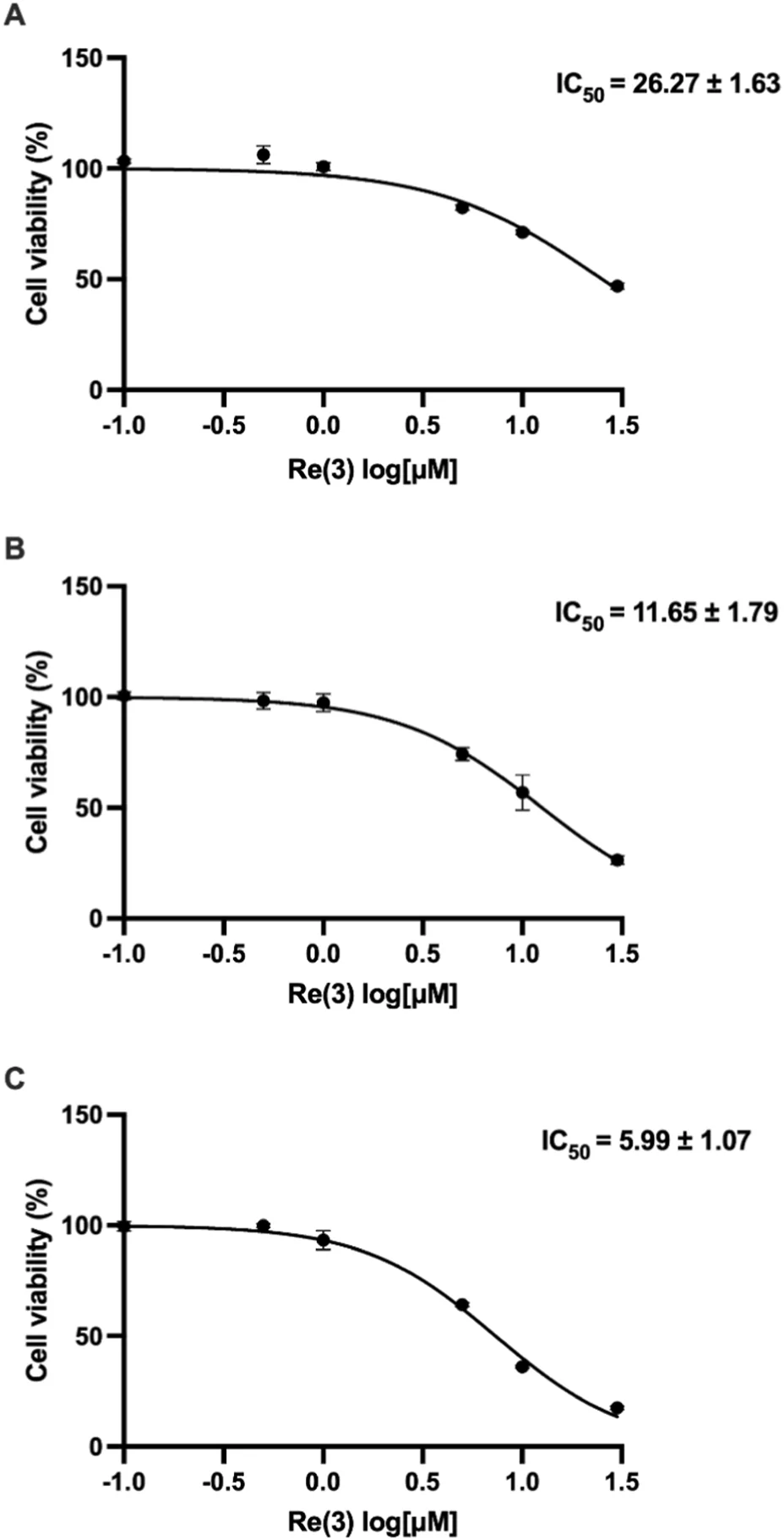
Concentration-response curve of Re(3) complex in HepG2 cells after 24 h **(A)**, 48 h **(B)**, and 72 h **(C)** incubation as determined with resazurin assay. Data shown are mean ± SEM of 3 independent experiments.

Furthermore, the cytotoxic effect of Re(3) was evident as early as 24 h post-treatment, suggesting a rapid cellular response, likely mediated by mitochondrial dysfunction and metabolic inhibition, as demonstrated by the resazurin assay (Grey et al., 2019). The significant difference in sensitivity between HepG2 and Vero cells implies a preferential interaction of the Re(3) complex with tumor-specific metabolic pathways or membrane transport systems. These findings collectively suggest that the Re(3) complex exerts selective antiproliferative effects on the HCC cells HepG2 while sparing the normal Vero cells, highlighting its potential as a therapeutic candidate for targeted HCC treatment.

### 3.3 Re(3) induces apoptosis on HepG2 cells

To quantitatively assess whether Re(3) tricarbonyl complex induces HepG2 cell apoptosis, cell death was analyzed using Annexin V-FITC/7-AAD double staining followed by flow cytometry. As shown in Figure 3, treatment with 5 μM Re(3) for 24 and 48 h produced a significant, time-dependent increase in both early (Annexin V^+^/7-AAD^-^) and late apoptotic (Annexin V^+^/7-AAD^+^) populations compared with untreated controls (*p* < 0.01 and *p* < 0.0001). After 48 h of exposure, more than 60% of the total cell population exhibited apoptotic features, confirming that cell death occurred primarily through apoptosis rather than necrosis.

**FIGURE 3:**
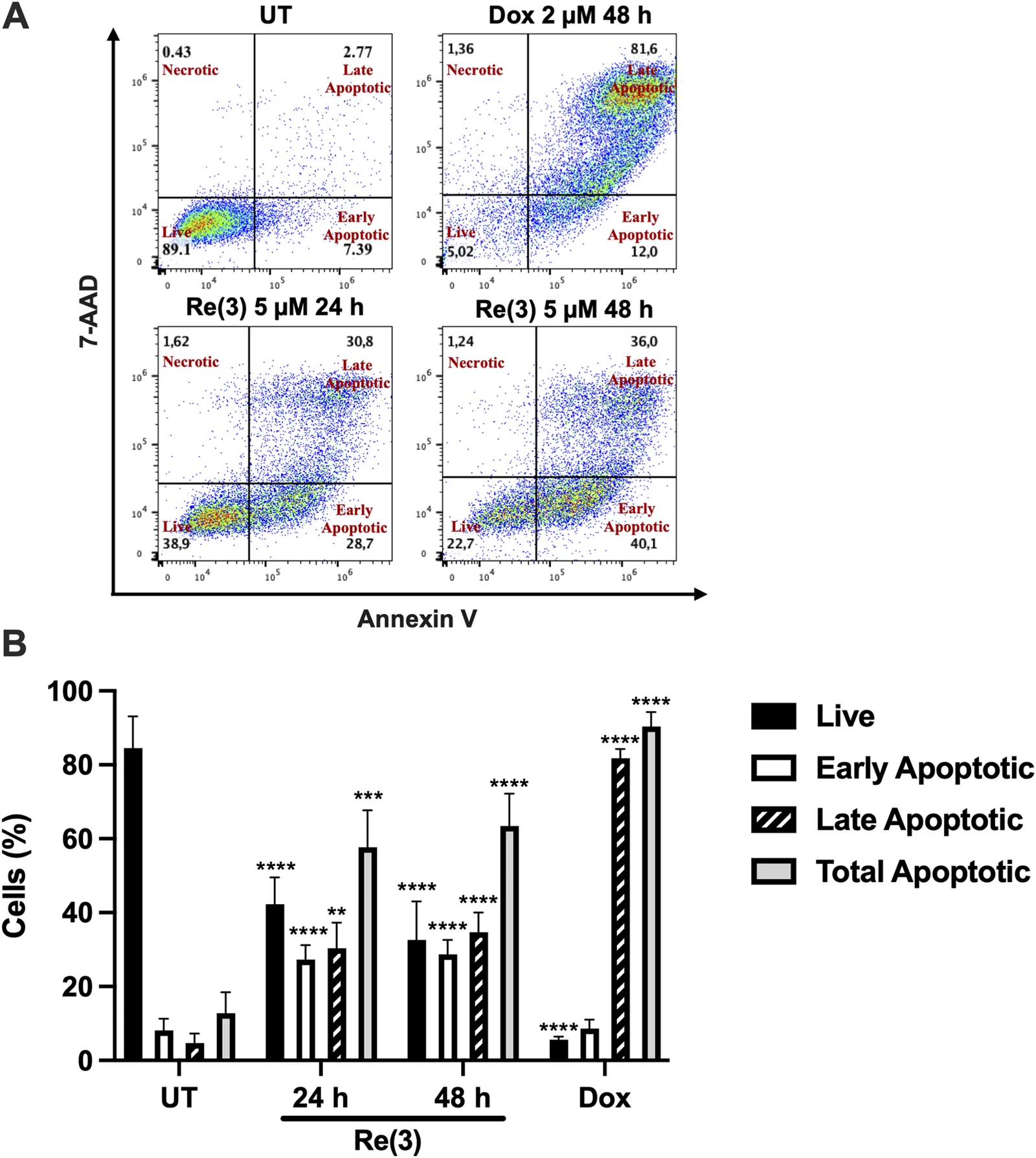
Re(3)-induced apoptosis in Hep-G2 cells as assayed by flow cytometry. **(A)** HepG2 cells were treated with Dox (2 μM) for 48 h and **Re(3)** (5 μM) for 24 and 48 h. The cells were then harvested and stained with Annexin V and 7-AAD, and flow cytometric analysis was performed to analyze apoptosis; **(B)** Summary of the apoptosis data in histogram form. Data are expressed as the mean ± standard error (SD) from at least three independent experiments. **p < 0.01 vs. the untreated (UT) group; ***p < 0.001 vs. the untreated group; ****p < 0.0001 vs. the untreated group.

The proportion of necrotic cells (Annexin V^−^/7-AAD^+^) remained minimal across all experimental conditions, indicating that Re(3) triggers a controlled, non-inflammatory mode of cell death. This selective apoptotic profile is consistent with the activation of caspase-3/7 and the modulation of pro- and anti-apoptotic proteins observed by western blotting. Moreover, the progressive shift from early to late apoptosis over time suggests sustained activation of the apoptotic machinery, likely driven by cumulative oxidative stress and mitochondrial disruption.

Quantitative analysis further revealed that Re(3)-treated HepG2 cells exhibited a nearly threefold increase in total apoptotic fraction compared to control cells. In contrast, non-tumor Vero cells displayed a negligible apoptotic response under the same conditions. This selective cytotoxicity reinforces the Re(3) complex’s preferential affinity for malignant cells, possibly due to differences in redox homeostasis and membrane transport mechanisms. Overall, the flow cytometry data confirm that the Re(3) complex effectively induces apoptosis in a dose- and time-dependent manner.

### 3.4 Re(3) induces apoptosis and caspase activation

Caspase-Glo® assays confirmed elevated caspase-3/7 activity after 4–16 h of Re(3) exposure (Figure 4). The activation of caspase-3/7, key executioner enzymes of apoptosis, suggests mitochondrial involvement and the release of cytochrome c as potential upstream events. Furthermore, the time-dependent increase in apoptotic cells indicates that Re(3) initiates a cascade of molecular events culminating in caspase activation and DNA degradation. This pro-apoptotic response, together with the previously observed selective cytotoxicity, suggests that the compound acts through specific signaling mechanisms that preferentially target tumor cell survival pathways.

**FIGURE 4:**
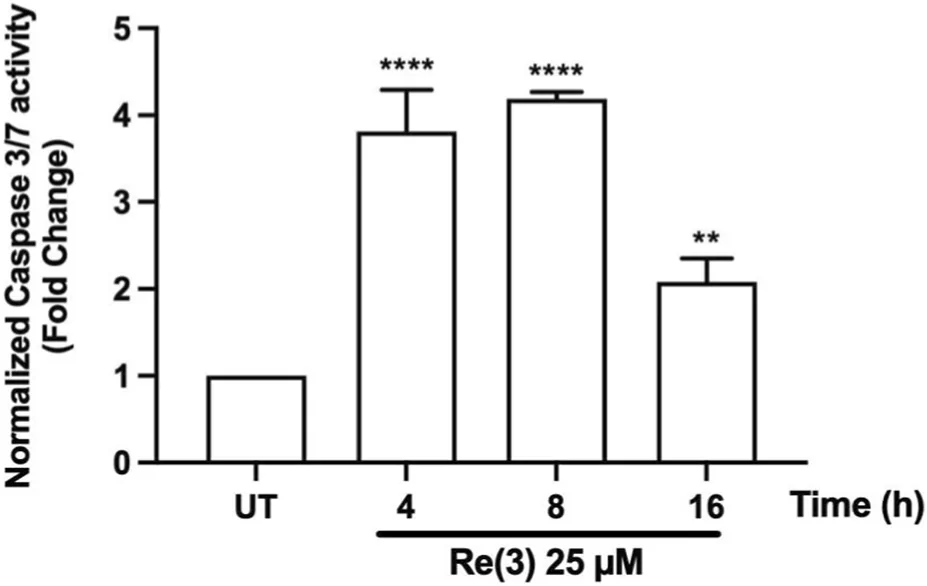
Normalized results of the enzymatic activity analysis of caspase3/7 proteins using the Caspase-Glo® kit in the Hep-G2 cell line. The results are expressed in RLU (Relative Light Units). Hep-G2 cells were stimulated with 25 µM of Re(3) at time points of 4, 8, and 16 h. Statistical differences were assessed by an ordinary one-way analysis of variance (ANOVA) followed by Dunnett´s *post hoc* test. **: *p* < 0.01 and ****: *p <* 0.0001 against the untreated condition. Graph bars show the mean ± SEM. (*n* = 3).

Notably, the observed caspase activation may be closely associated with oxidative stress in cells, as redox imbalance is a recognized trigger of mitochondria-mediated apoptosis. Given the redox-active nature of rhenium complexes, subsequent analyses were directed toward evaluating whether intracellular ROS generation contributes to the cytotoxic and apoptotic mechanisms induced by Re(3).

### 3.5 Re(3) increases intracellular ROS generation

To investigate whether the Re(3) tricarbonyl complex may influence the intracellular oxidative stress of HepG2, along with the cytotoxic and apoptotic effects of the Re(3), total intracellular ROS levels were quantified using the DCFH-DA fluorescence assay. Exposure of HepG2 cells to increasing concentrations of Re(3) (1–20 μM) resulted in a significant, dose-dependent increase in total intracellular ROS levels at 2 and 4 h of incubation (*p* < 0.001 compared with untreated controls; Figure 5). The magnitude of ROS production was comparable to that induced by the CuSO_4_ positive control, confirming the compound’s strong oxidative potential.

**FIGURE 5:**
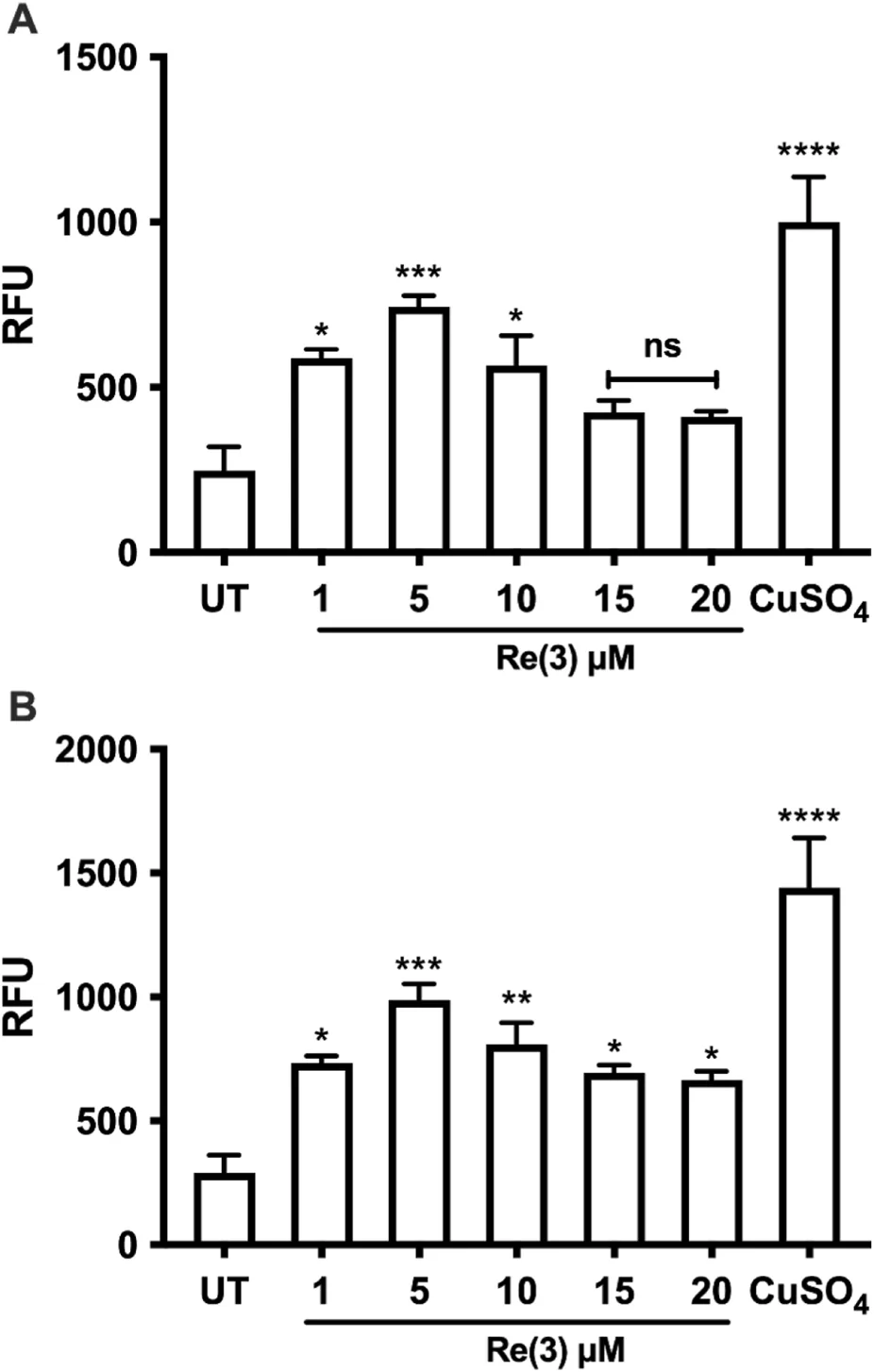
Effects of Re(3) on HepG2 cells intracellular ROS generation. Cells were exposed to Re(3) at 1–20 *μ*M for 2 h **(A)** and 4 h **(B)**. CuSO_4_ is used as a positive control. Stained cells with DCFDA were analyzed by a fluorescence plate reader. Data are expressed as the mean ± SEM from three independent experiments, each performed in triplicate. A one-way ANOVA (Kruskal–Wallis) assessed statistical differences, followed by Dunn’s *post hoc* test. *p < 0.05; **p < 0.01; ***p < 0.001 ****p < 0.0001 vs. Untreated (UT) group. The bar scale represents 20 μM.

These findings indicate that the Re(3) complex promotes an early redox imbalance in tumor cells, likely via disruption of the mitochondrial electron transport chain or through redox cycling reactions involving the rhenium center. The accumulation of total intracellular ROS levels can initiate oxidative damage to cellular macromolecules, including lipids, proteins, and DNA, contributing to the loss of mitochondrial membrane integrity and activation of the intrinsic apoptotic pathway. The temporal correlation between ROS accumulation and caspase activation suggests a mechanistic link in which oxidative stress functions as the key upstream signal for apoptosis induction.

Oxidative stress response underscores the potential of rhenium-based complexes as dual-function agents that can induce both redox-mediated cytotoxicity and caspase-dependent apoptosis in HCC.

### 3.6 Re(3) on the expression of apoptotic markers

Western blot analysis was performed to assess the expression of key apoptotic proteins following treatment of HepG2 cells with Re(3)-tricarbonyl complex. As shown in Figure 6, a time-dependent modulation of both pro-apoptotic and anti-apoptotic markers was observed after exposure to 20 μM Re(3) for 0.5–8 h. Notably, a marked upregulation of caspase-3, caspase-9, and cytochrome c was observed, consistent with activation of the intrinsic (mitochondrial) apoptotic pathway. Re(3) complex has no extrinsic (death receptor-mediated) activity for caspase-8. Concurrently, the expression of the anti-apoptotic protein Bcl-XL was significantly downregulated (*p* < 0.01), suggesting that the compound disrupts mitochondrial homeostasis and promotes cell death signaling.

**FIGURE 6:**
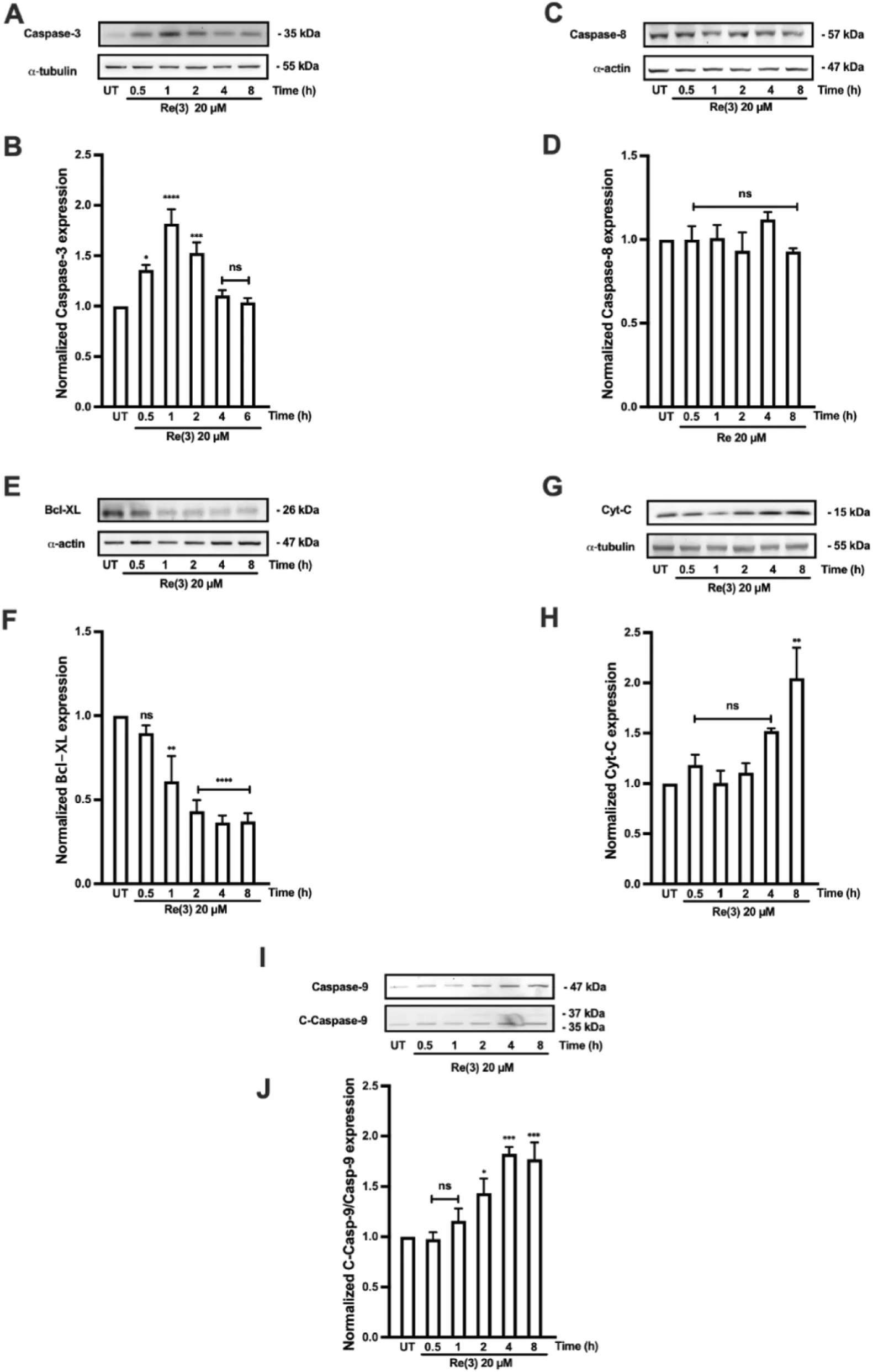
Re(3) induced changes in the expression of pro-apoptotic and anti-apoptotic markers. **(A–J)** Hep-G2 cells were exposed to tricarbonyl rhenium complex (20 μM) for 0, 0,5, 1, 2, 4, and 8 h, and protein expression was analyzed (**A,C,E,G,I)**. Representative images from western blot experiments performed for the detection of antiapoptotic markers Bcl-XL **(A)** and proapoptotic markers caspase-3 **(C)**, caspase-8 **(E)**, cytochrome-c **(G)**, and caspase-9 **(I)**. Densitometric analyses of the experiments shown in (**B,D,F,H,J)**. Protein levels were normalized to tubulin and actin levels. Data are expressed relative to the untreated condition. Statistical differences were assessed using an ordinary one-way analysis of variance (ANOVA) followed by Dunnett´s *post hoc* test. *: *p* < 0.05, **: *p* < 0.01, ***: p < 0.001 and ****: *p <* 0.0001 against the untreated condition. Graph bars represent the mean ± SEM of three independent experiments (*n* = 3).

The release of cytochrome c from the mitochondria into the cytosol and the subsequent activation of caspase-9 strongly support the involvement of the intrinsic apoptotic cascade. Activation of caspase-8 further indicates crosstalk between the extrinsic and intrinsic pathways, amplifying apoptotic signaling within the cell. This dual activation profile reflects a complex molecular mechanism in which the Re(3) complex induces apoptosis via multiple converging routes rather than a single, linear pathway. Densitometric analyses confirmed that cleaved caspase-3 and cytochrome c expression increased significantly within 4–8 h post-treatment, temporally corresponding to ROS generation and phospho-p53 accumulation observed by immunofluorescence assays. These results suggest that oxidative stress is a primary trigger of p53 phosphorylation, mitochondrial membrane permeabilization, and subsequent caspase-dependent apoptosis.

Collectively, these molecular changes indicate that the Re(3) complex initiates a coordinated apoptotic program involving oxidative stress, mitochondrial dysfunction, and suppression of antiapoptotic defenses. This multifaceted mechanism underscores the therapeutic potential of this targeted metal-based anticancer agent, which exhibits high selectivity for hepatocellular carcinoma cells.

### 3.7 The Re(3)-complex increased the expression of phosphorylated (p)-p53 and caspase-3, along with a reduction in Bcl-XL

To further elucidate the subcellular mechanisms involved in the pro-apoptotic activity of the Re(3) tricarbonyl complex, immunofluorescence microscopy analysis was performed on HepG2 cells treated with 10 μM Re(3) for 24 h. As shown in Figure 7, the treated cells exhibited significant alterations in the distribution and expression of apoptosis-related markers, including phosphorylated (p)-p53, caspase-3, Bcl-XL, and α-tubulin, with cytoskeletal rearrangement. Compared with the untreated controls, Re(3)-treated cells showed increased p-p53 and caspase-3 immunostaining, confirming apoptosis at the cellular level.

**FIGURE 7:**
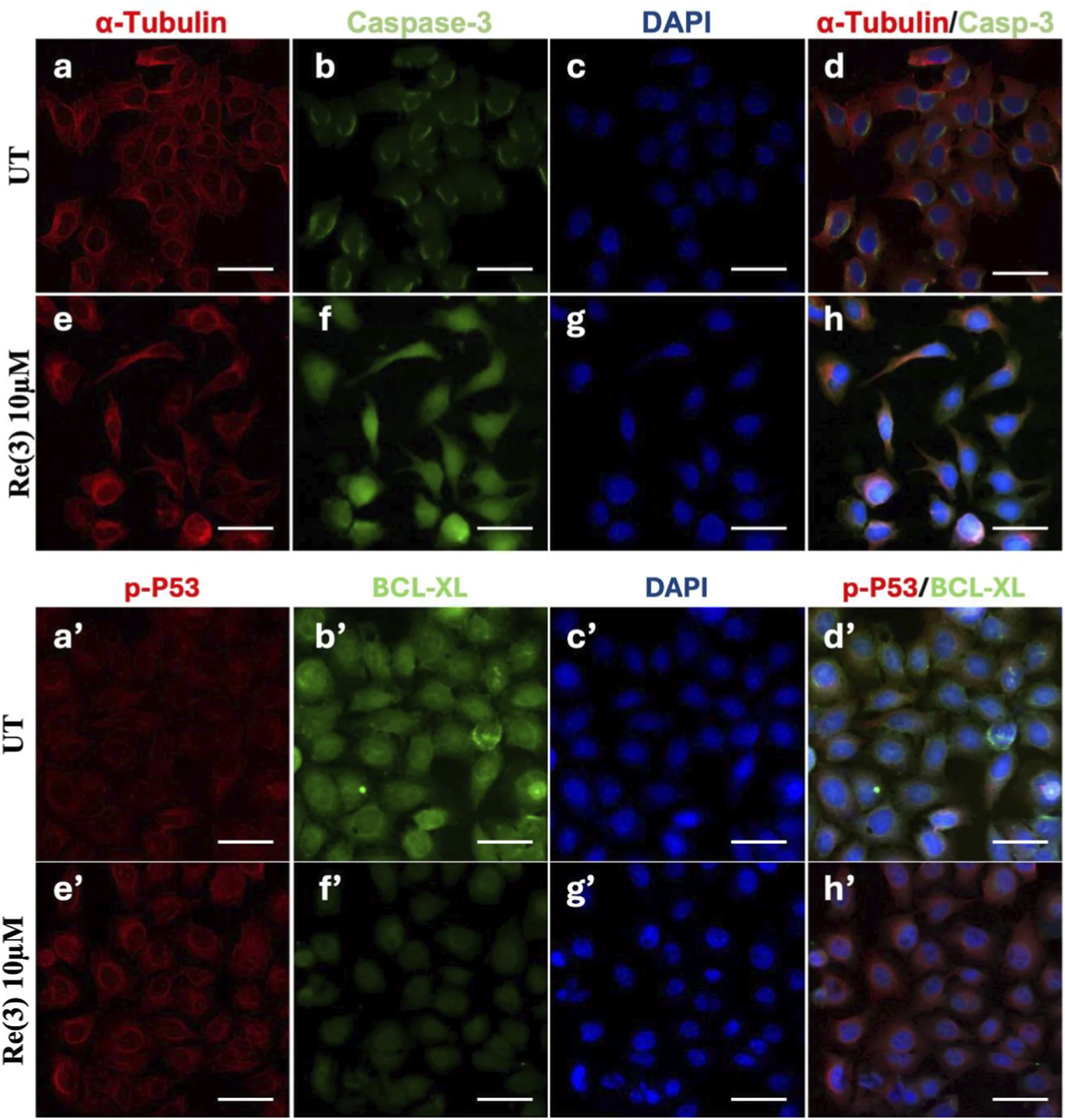
Cellular distribution of epithelial hepatocarcinoma cells and fibrotic markers involved in Re(3) induced apoptosis of Hep-G2. (**a–h**) Representative images from Hep-G2 in the absence of Re(3) (0 *μ*M Re(3) (**a–d, a’–d’**) or in the presence of 10 *μ*M Re(3) (**e–h,e’–h’**), for 24 h α-Tubulin and p-P53 (red), and Caspase-3 and Bcl-XL (green) were detected, respectively. The nuclei were stained with DAPI. The bar scale represents 10 *μ*m (*n* = 3).

The observed increase in p-p53 levels suggests that the Re(3) complex may trigger p53-dependent stress responses, possibly through DNA damage or oxidative signaling pathways. The simultaneous loss of Bcl-XL fluorescence indicates the disruption of mitochondrial membrane integrity and suppression of anti-apoptotic defenses, further supporting the activation of the intrinsic apoptotic pathway. In addition, cytoskeletal rearrangements were evident, as indicated by altered α-tubulin organization and cell rounding, which are typical features of apoptotic cells.

These immunofluorescent observations corroborate the biochemical data obtained from caspase activity assays and western blot analyses, demonstrating that Re(3) treatment induces a coordinated apoptotic response involving both nuclear and mitochondrial compartments. The distinct localization of active caspase-3 and p-p53 underscores the compound’s ability to modulate multiple signaling axes simultaneously, leading to irreversible commitment to apoptosis.

Taken together, these immunofluorescence findings confirm that the Re(3) complex exerts its cytotoxic effects through mitochondrial destabilization, caspase activation, and p53-mediated apoptosis. This dual localization pattern supports the hypothesis that the compound acts through a multifactorial mechanism, integrating oxidative stress signaling, DNA damage response, and mitochondrial dysfunction, to selectively induce death in hepatocellular carcinoma cells.

These findings consolidate the evidence that the compound exerts antitumor effects through a multifactorial mechanism involving oxidative stress, mitochondrial damage, and caspase-mediated apoptosis, establishing a strong foundation for subsequent *in vivo* investigations.

## 4 Discussion

The present study demonstrates that the Re(3) tricarbonyl complex exhibits potent and selective cytotoxic effects against HepG2 cells, with minimal toxicity to non-tumor Vero cells. The dose-dependent reduction in cell viability, accompanied by caspase activation, ROS generation, and mitochondrial dysfunction, confirmed that the compound’s cytotoxic mechanism primarily involves inducing apoptosis via both intrinsic and extrinsic pathways. These findings are consistent with the growing evidence that rhenium-based organometallic complexes are a promising class of chemotherapeutic agents capable of targeting tumor cells via multiple molecular routes (Leonidova and Gasser, 2014).

The activation of caspase-3/7, caspase-8, and caspase-9, along with the observed downregulation of Bcl-XL, indicates that the Re(3) complex triggers both mitochondrial and receptor-mediated apoptotic processes. The concomitant release of cytochrome c and increase in p-p53 levels further support the involvement of Re(3) in triggering apoptosis through the intrinsic pathway, possibly involving mitochondrial modulation. These results are consistent with previous studies, showing that Re(I) tricarbonyl complexes can induce apoptosis via mitochondrial destabilization and oxidative stress in multiple tumor cell types.

A notable observation in this study was the significant increase in total intracellular ROS levels following treatment with the Re(3) complex. Elevated total intracellular ROS levels are a hallmark of rhenium-mediated cytotoxicity and often precede caspase activation and DNA fragmentation. Excessive oxidative stress can compromise the mitochondrial membrane potential, leading to cytochrome c release and subsequent activation of the caspase cascade. This mechanism aligns with the well-established redox-active nature of rhenium complexes, in which the metal center can participate in electron-transfer reactions that generate reactive intermediates. The greater ROS accumulation in HepG2 cells likely reflects differences in basal metabolic rate and antioxidant defense capacity, highlighting the compound’s preferential cytotoxicity toward cancerous cells. Future studies could employ selective probes, such as MitoSOX for mitochondrial superoxide and Amplex Red for hydrogen peroxide, to facilitate further investigation.

Structurally, the 5-chloro-1,10-phenanthroline ligand (Clphen) plays a crucial role in modulating biological activity. Substitution of the phenanthroline ring enhances lipophilicity and cellular uptake, promoting mitochondrial localization and improving interactions with DNA and proteins (Frin and Murakami Iha, 2011). Previous studies have demonstrated that electron-withdrawing substituents, such as chlorine, can stabilize Re–ligand charge-transfer states and increase photophysical reactivity (Świtlicka et al., 2016). This may have contributed to the strong ROS production and enhanced pro-apoptotic signaling observed in our experiments.

A counterintuitive yet crucial finding from our computational study concerns the behavior of the Chemical Hardness (η), defined as half of the HOMO-LUMO gap. Generally, higher reactivity (smaller gap) favors biological activity. However, our data reveal that the active complex Re(3) possesses the highest chemical hardness (η = 2.37 eV) in the series, surpassing both the inactive amino (η = 2.22 eV) and nitro (η = 1.6 eV) complexes. This result suggests that the efficacy of Re(3) depends not only on its thermodynamic capacity to accept electrons (electrophilicity) but also on its kinetic stability. The nitro complex (Re(2)), despite exhibiting massive electrophilicity, presents a dangerously small energy gap (1.66 eV). This makes it a chemically “soft” and highly reactive species, likely prone to non-specific side reactions, rapid decomposition, or metabolic detoxification. The limitations of gas-phase DFT calculations highlight the need for experimental evaluation of intracellular stability, protein binding, and pharmacokinetic behavior.

In contrast, Re(3) with the chlorinated ligand stabilizes the LUMO sufficiently to enable ROS generation (high electrophilicity); however, the complex maintains a wide energy gap that confers resistance against spurious cytosolic nucleophiles. This specific combination of a favorable redox potential with high kinetic stability explains the superiority of the chloro-substituted design, creating a “tuned electrophile” capable of surviving cellular transport and acting selectively within the mitochondria.

The simultaneous activation of p53 and suppression of Bcl-XL suggests that Re(3) modulates both the transcriptional and mitochondrial apoptotic pathways. Phosphorylation and nuclear accumulation of p53 imply a DNA damage or oxidative stress response, leading to the transcriptional activation of downstream pro-apoptotic genes, such as *Bax* and *PUMA*. This dual mechanism reinforces the potential of this compound as a multitarget agent capable of overcoming the resistance mechanisms commonly associated with conventional chemotherapy (Delasoie et al., 2020). Nevertheless, further analyses are needed to verify this mechanism for Re(3) in HepG2 cells.

Notably, the Re(3) complex demonstrated selectivity toward hepatocellular carcinoma HepG2 cells, sparing the non-malignant cells even at higher concentrations. This selective toxicity could result from differences in metal uptake and intracellular processing between normal and transformed cells, as tumor cells often exhibit enhanced permeability, increased mitochondrial activity, and disrupted redox balance. Similar trends have been reported for other Re(I) complexes that display high tumor specificity and minimal systemic toxicity in *in vivo* models (Konkankit et al., 2019). Although Re(3) demonstrated potential for selective toxicity in HepG2 cells, this finding is limited to a single cell line. Given the well-documented molecular heterogeneity of hepatocellular carcinoma, future studies should validate these findings in Huh7, Hep3B, and PLC/PRF/5 cells, as well as in primary HCC models, before any generalization across HCC subtypes can be made.

In summary, our results suggest that Re(3) exerts its anticancer activity through a multifactorial mechanism involving (i) generation of oxidative stress, (ii) mitochondrial modulation and release of cytochrome c, (iii) activation of caspase cascades, and (iv) modulation of p53-mediated apoptotic signaling (Figure 8). The combination of these effects results in potent and selective cytotoxicity against hepatocellular carcinoma cells. The integrated photophysical and biochemical properties of this complex highlight the potential of halogenated phenanthroline-based rhenium compounds as next-generation metal-based chemotherapeutics for liver cancer treatment.

**FIGURE 8:**
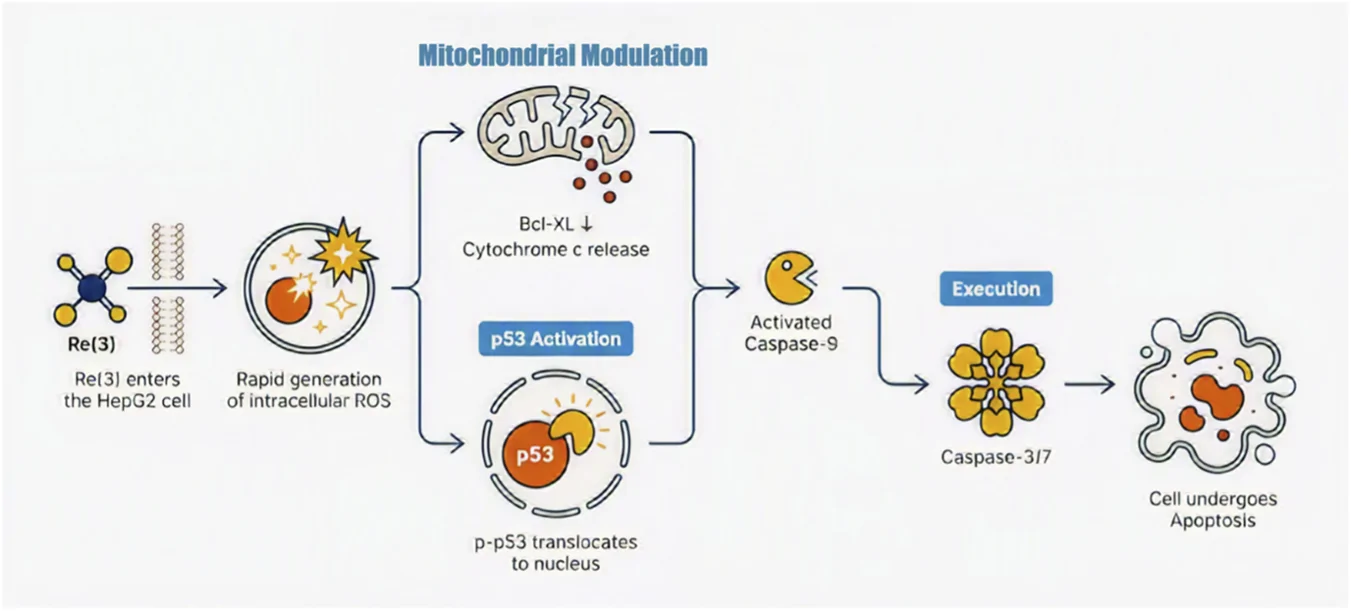
Proposed model for Re(3)-mediated apoptosis in human hepatoma cells.

Although the present results support a relationship between the electronic properties of the Re(I) complexes and their biological activity, additional factors may also contribute to the observed differences in cytotoxicity. In particular, chloride-to-water substitution (aquation) has been recognized as an important activation process in several classes of metal-based anticancer agents and may influence the solution speciation and biological behavior of *fac*-[Re(CO)_3_(NN)Cl] complexes (Knopf et al., 2017). Therefore, aquation kinetics could contribute, together with the electronic effects discussed herein, to the observed structure–activity relationships (Alessio and Messori, 2019). Since aquation was not investigated in the present study, its contribution remains to be established in future mechanistic studies.

Future studies should aim to evaluate the *in vivo* pharmacokinetics, biodistribution, and therapeutic efficacy of this complex, as well as its potential synergy with established chemotherapeutic or photodynamic agents. Elucidating the molecular targets and binding interactions at the mitochondrial and nuclear levels will further elucidate the precise mechanisms underlying its selective anticancer activity.

## 5 Conclusion

Re(3) tricarbonyl complex fac-[Re(CO)3(Clphen)Cl] demonstrated potent and selective cytotoxic activity against hepatocellular carcinoma (HepG2) cells while exhibiting minimal toxicity toward normal Vero cells. The compound induced apoptosis through a multifaceted mechanism involving oxidative stress, mitochondrial dysfunction, and activation of both intrinsic and extrinsic caspase-dependent pathways. Increased total intracellular ROS levels, cytochrome c release, and upregulation of phosphorylated p53, together with downregulation of Bcl-XL, collectively highlight its ability to disrupt redox balance and selectively trigger programmed cell death in tumor cells.

These findings position Re(3) tricarbonyl complex *fac*-[Re(CO)_3_(Clphen)Cl] as a promising prototype for the development of rhenium-based chemotherapeutic agents targeting hepatocellular carcinoma. Its selective cytotoxicity, redox-mediated mechanism, and favorable biological profile highlight its potential as a lead compound for further *in vivo* testing and rational design of multifunctional anticancer agent complexes.

## Data Availability

The original contributions presented in the study are included in the article/Supplementary Material, further inquiries can be directed to the corresponding authors.
