# Supplementary material for: Rhenium(I) tricarbonyl complexes with 1,10-phenanthroline derivatives: potential anticancer agents for hepatocellular carcinoma
Source: Front Pharmacol. 2026 Sep 21;17:1926840. doi: 10.3389/fphar.2026.1926840 (PMC13635290; doi:10.3389/fphar.2026.1926840)
Supplement: Supplementary file 1 [file Supplementaryfile1.docx]

**SUPPLEMENTARY MATERIAL**

**Rhenium(I) Tricarbonyl Complexes with 1,10-Phenanthroline Derivatives: Potential Anticancer Agents for Hepatocellular Carcinoma**

César Zúñiga^1,2^, Diego Oyarzún^3^, Carlos Cardoso^4^, Valentina Melo^4^, Ignacio Montorfano^1,2^, Yolanda Prado^5,6^, Felipe Simon^5,6^, Juan F. Santibáñez^7,8^, Rodrigo Ramírez-Tagle^9^, Javier Echeverría^10*^, César Echeverría^1,2*^

^1^Instituto de Ciencias Naturales, Facultad de Medicina Veterinaria y Agronomía, Universidad de Las Américas, Santiago, Chile.

^2^Centro de Investigación en Ciencias Biológicas y Químicas, Universidad de Las Américas, Santiago, Chile.

^3^Departamento de Química y Biología, Facultad de Ciencias Naturales, Universidad de Atacama, Copiapó, Chile.

^4^ATACAMA-OMICS, Laboratorio de Biología Molecular y Genómica, Facultad de Medicina, Universidad de Atacama, Copiapó, Chile.

^5^Center for Research on Pandemic Resilience, Faculty of Life Sciences and Institute of Public Health, Universidad Andres Bello, Santiago, Chile.

^6^Millennium Institute on Immunology and Immunotherapy, Santiago, Chile.

^7^Centro Integrativo de Biología y Química Aplicada (CIBQA), Universidad Bernardo O'Higgins, 8370993 Santiago, Chile.

^8^Group for Molecular Oncology, University of Belgrade, Institute for Medical Research, National Institute of Republic of Serbia, Belgrade, Serbia.

^9^Facultad de Ingeniería y Arquitectura, Universidad Central de Chile, Santiago, Chile.

^10^Departamento de Ciencias del Ambiente, Facultad de Química y Biología, Universidad de Santiago de Chile, Santiago, Chile.

*****Authors to whom correspondence should be addressed; e-mails: javier.echeverriam@usach.cl; [cecheverria@udla.cl](about:blank)

**Index**

**Figure S1.** ^1^H NMR spectrum of complex *fac*-Re(CO)_3_(PhenNH_2_)Cl **Re(1)**..……………………………………………………..page 3

**Figure S2.** ^1^H NMR spectrum of complex *fac*-Re(CO)_3_(PhenNO_2_)Cl **Re(2)**.……………………………………………………...page 4

**Figure S3.** ^1^H NMR spectrum of complex *fac*-Re(CO)_3_(PhenCl)Cl **Re(3)**……………………………………………….…...........page 5

**Figure S4**. FT-IR spectrum of complex *fac-*[Re(CO)_3_(PhenNH_2_)Cl] **Re(1)**…..……………………………………………………page 6

**Figure** **S5**. FT-IR spectrum of complex *fac-*[Re(CO)_3_(PhenNO_2_)Cl **Re(2)**…..…………………………………………………….page 7

**Figure S6**. FT-IR spectrum of complex *fac-*[Re(CO)_3_(PhenCl)Cl] **Re(3)**……………………………………………………….....page 8

**Figure S7.** Molecular orbital diagrams of the examined rhenium complexes………….…………………………………….…......page 9

**Table S1.** Primary and secondary antibodies used in western blot experiments…………….………………………………..…….page 7

**Table S2.** Primary and secondary antibodies used in immunocytochemistry……………….………………………………….…..page 8

**Figure S1.** ^1^H NMR spectrum of complex *fac*-Re(CO)_3_(PhenNH_2_)Cl **Re(1)**.

**Figure S2.** ^1^H NMR spectrum of complex *fac*-Re(CO)_3_(PhenNO_2_)Cl **Re(2)**.

**Figure S3.** ^1^H NMR spectrum of complex *fac*-Re(CO)_3_(PhenCl)Cl **Re(3)**.

**Figure S4**: FT-IR spectrum of complex *fac-*[Re(CO)_3_(PhenNH_2_)Cl

**Figure** **S5**: FT-IR spectrum of complex *fac-*[Re(CO)_3_(PhenNO_2_)Cl **Re(2)**

**Figure S6**: FT-IR spectrum of complex *fac-*[Re(CO)_3_(PhenCl)Cl] **Re(3)**

|  | **LUMO +1** | **LUMO** | **HOMO** | **HOMO-1** |
| --- | --- | --- | --- | --- |
| **Re(1)** |  |  |  |  |
| **Re(2)** |  |  |  |  |
| **Re(3)** |  |  |  |  |

**Figure S7.** Molecular orbital diagrams of the examined rhenium complexes

**Table S1.** Primary and secondary antibodies used in western blot experiments.

| **Primary Ab** | **Dilution** | **Source** | **Incubation**  **time** | **Incubation**  **temperature** | **Brand** |
| --- | --- | --- | --- | --- | --- |
| Caspase-3 | 1:1000 | Rabbit | 2 h | RT | Cell Signaling, MA, USA |
| Caspase-9 | 1:1000 | Rabbit | 2 h | RT | Cell Signaling, MA, USA |
| Caspase-8 | 1:1000 | Mouse | 2 h | RT | Cell Signaling, MA, USA |
| α-Tubulin | 1:5000 | Mouse | 2 h | RT | Sigma-Aldrich, MO, USA |
| a-Actin | 1:5000 | Mouse | 2 h | RT | Sigma-Aldrich, MO, USA |
| Cyt-C | 1:1000 | Rabbit | 2 h | RT | Abcam, Cambridge, UK |
| Bcl-XL | 1:1000 | Rabbit | 2 h | RT | Cell Signaling, MA, USA |

| **Secondary Ab** | **Dilution** | **Source** | **Incubation**  **time** | **Incubation**  **temperature** | **Brand** |
| --- | --- | --- | --- | --- | --- |
| Anti- Mouse HRP | 1:5000 | Goat | 2 h | RT | R&D Systems |
| Anti- Rabbit HRP | 1:5000 | Goat | 2 h | RT | Sigma-Aldrich |

**Ab: antibody, RT: room temperature.**

**Table S2.** Primary and secondary antibodies used in immunocytochemistry.

| **Primary Ab** | **Dilution** | **Source** | **Incubation**  **time** | **Incubation**  **temperature** | **Brand** |
| --- | --- | --- | --- | --- | --- |
| Bcl-XL | 1:100 | Rabbit | 2 h | 4ºC | Santa Cruz, CA, USA Santa Cruz, CA, USA |
| p-P53 | 1:100 | Mouse | 2 h | 4ºC | Cell Signaling, MA, USA |
| Caspase-3 | 1:100 | Rabbit | 2 h | 4ºC | Cell Signaling, MA, USA |
| α-Tubulin | 1:500 | Mouse | 2 h | 4ºC | Sigma-Aldrich, MO, USA |

| **Secondary Ab** | **Dilution** | **Source** | **Incubation**  **time** | **Incubation**  **temperature** | **Brand** |
| --- | --- | --- | --- | --- | --- |
| Anti- Rabbit Alexa-594 | 1:250 | Goat | 2 h | 4ºC | Invitrogen, CA, USA  USA |
| Anti- Mouse Alexa-488 | 1:250 | Goat | 2 h | 4ºC | Invitrogen, CA, USA |

**Ab: antibody.**
